# An Energy Detection Algorithm with Clustering-Based False Alarm Suppression for Magnetic Anomaly Detection

**DOI:** 10.3390/s26051627

**Published:** 2026-03-05

**Authors:** Jinghua Yu, Changping Du, Xiang Peng

**Affiliations:** School of Electronics, Peking University, Beijing 100871, China; 2101111413@stu.pku.edu.cn (J.Y.); dcp@pku.edu.cn (C.D.)

**Keywords:** magnetic anomaly detection, hierarchical clustering, optimal cut height, Orthogonal Basis Function (OBF), Constant False Alarm Rate (CFAR)

## Abstract

To overcome the limitations of Orthonormal Basis Function (OBF) methods in magnetic anomaly detection, including high false alarm rates and ambiguous target localization due to background noise, this paper introduces a high-confidence detection algorithm based on hierarchical clustering with an optimal cut height. The core of our approach is a theoretically derived optimal cut height, which is calculated from a physical model of the magnetic dipole’s vertical gradient field. This model establishes the implicit functional relationship between the effective detection range and key parameters, including magnetic moment orientation, geomagnetic inclination, and sensor height. The calculated optimal cut height serves as the critical criterion in a complete-linkage hierarchical clustering algorithm, which processes the alarm point clouds generated by a preliminary Greatest-of Cell-Averaging Constant False Alarm Rate (GOCA-CFAR) detector. This effectively suppresses isolated false alarms caused by background fluctuations while preserving spatially coherent alarm clusters within the target’s effective detection range, thereby significantly enhancing detection confidence. Results from both simulations and field experiments validate the efficacy of the proposed algorithm, demonstrating its superior capability to reliably discriminate genuine targets from false alarms compared to traditional one-dimensional CFAR detection.

## 1. Introduction

Magnetic Anomaly Detection (MAD) is a passive detection technology based on the characteristics of geomagnetic anomaly. It offers advantages such as non-active probing, high efficiency, and non-invasiveness, and is often used as a supplementary method to optical detection, radar detection, and thermal inspection. It is widely applied in mineral resource exploration [1,2], underwater target localization [3,4], archaeological research [5,6], and unexploded ordnance detection [7,8,9]. However, in many of these real-world scenarios, the practical utility of MAD is severely compromised not by a lack of sensitivity, but by an overabundance of alarms—a significant portion of which are false positives induced by environmental and geological noise. These false alarms lead to substantial costs and time consumption [10]. Therefore, the core challenge in the field has expanded from mere detection to the accurate identification of true targets.

Advances in optically pumped magnetometers, known for their high sensitivity (down to picotesla levels) and accuracy, have made them essential for magnetic anomaly detection. However, this high sensitivity also makes them susceptible to environmental noise, demanding more sophisticated algorithms that can reliably distinguish targets from false alarms. To use the high-sensitivity magnetic data, the Orthonormal Basis Function (OBF) decomposition method is widely employed owing to its high detection probability. This technique effectively extracts weak magnetic anomaly signals from background noise by constructing basis functions that match the target signal’s characteristics, demonstrating particularly strong performance in moving target detection [11,12,13]. To mitigate detection errors caused by unknown target motion states, Wang et al. proposed an adaptive OBF method that dynamically adjusts basis function parameters, increasing the accuracy of aligning the signal energy peak with the target’s closest point of approach by 54.1% [14]. Zhang et al. further enhanced the algorithm’s robustness against non-Gaussian noise by integrating OBF with wavelet transform, proposing a wavelet-domain OBF decomposition algorithm [15]. Kolster et al. proposed the Recursive Difference Inversion (RDI) method based on the point-dipole model, which can directly invert the recursive differences of UAV-based scalar magnetic survey data, addressing the challenges of dynamic sensor positions and complex preprocessing in Unexploded Ordnance (UXO) detection, but suffers from a high false alarm rate due to its inability to distinguish UXO from other magnetic targets [16]. Du et al. employed a method combining improved orthogonal basis functions with an optimized sparrow search algorithm to achieve high-precision magnetic anomaly localization of unexploded ordnance, reducing the best localization error to 0.2 m [17]. However, the studies indicate that while the OBF detector performs excellently in Gaussian white noise environments, its performance in practical geomagnetic noise (typically non-Gaussian) is highly dependent on pre-whitening filtration and degrades significantly without it [18]. Moreover, even with successful detection, traditional one-dimensional approaches struggle with ambiguous target localization and cannot systematically distinguish spatially coherent target signatures from random false alarms across multiple survey lines. On the other hand, the inversion algorithms based on magnetic gradient tensors are designed to accurately determine the spatial position of a target source [19,20]. However, their accuracy can be susceptible to noise and often relies on the assumption of a uniform background field. Furthermore, since these localization algorithms are applied after the detection stage, their performance is intrinsically linked to its output. A significant number of false alarms from the detector can thus reduce the efficiency of the localization process and affect the reliability of its results.

To address these interconnected challenges of false alarm suppression and spatial localization, this paper proposes a novel two-stage magnetic anomaly detection framework. The first stage employs OBF-CFAR detection to identify potential anomaly signals along individual survey lines, providing high sensitivity to weak magnetic signals. The second stage introduces hierarchical clustering with a physics-based optimal cut height to suppress false alarms by exploiting the spatial coherence of genuine magnetic anomalies. This spatial reasoning mechanism leverages the fundamental physical characteristic that real targets produce spatially clustered anomaly patterns within their effective magnetic influence range, whereas false alarms typically manifest as isolated, random detections.

The core innovation of our approach lies in replacing the empirical parameter selection that has traditionally limited clustering methods with a theoretically derived optimal cut height based on the physical properties of magnetic dipole fields. By establishing a vertical magnetic gradient field model and deriving the implicit functional relationship between the effective detection range and parameters such as magnetic moment orientation, geomagnetic inclination, and sensor height, we construct a theoretical model for calculating the optimal cut height based on extremum conditions. This physics-based parameter selection ensures that all alarm points generated by a genuine target are aggregated into a single cluster while effectively excluding spatially isolated false alarms, thereby overcoming the key limitation of traditional one-dimensional CFAR detection.

The main contributions of this work are:

- A comprehensive two-stage detection framework that integrates OBF-CFAR preliminary detection with hierarchical clustering for false alarm suppression, addressing both sensitivity and specificity requirements in practical MAD applications.
- A novel theoretical model that calculates the optimal cut height for hierarchical clustering directly from physical parameters, eliminating empirical guesswork. This is achieved by deriving the implicit functional relationship between the effective detection range and key variables including magnetic moment orientation, geomagnetic inclination, and sensor height.
- Extensive validation through both simulation and field experiments demonstrating significant improvements in false alarm suppression while maintaining high detection sensitivity.

**Figure 1:**
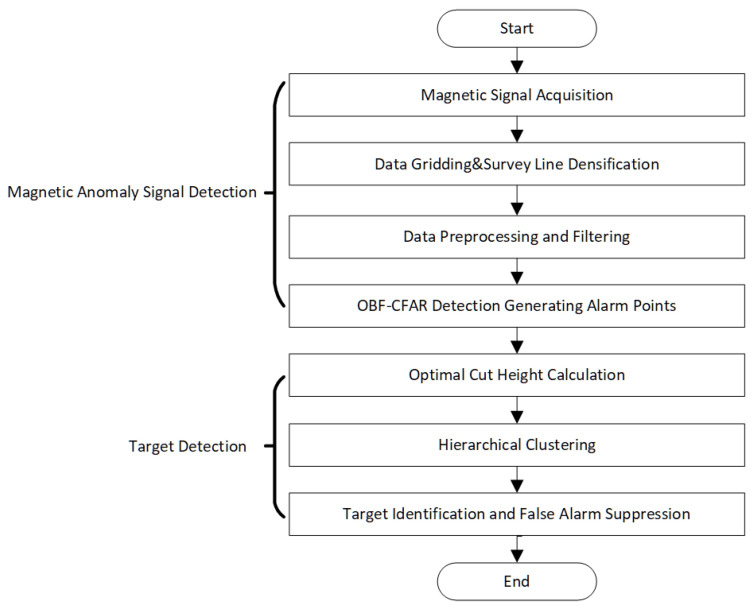
Magnetic anomaly detection flowchart.

The complete workflow, illustrated in Figure 1, encompasses magnetic anomaly signal detection using OBF-CFAR followed by target detection through spatial clustering with optimal cut height. Both simulation and field experiments demonstrate the algorithm’s effectiveness.

The remainder of this paper is organized as follows: Section 2 details the signal detection and clustering methodology. Section 3 presents simulation results validating the optimal cut height model. Section 4 describes field experiments and performance analysis. Finally, Section 5 concludes the paper.

## 2. Signal Detection and Clustering Methodology

This section details the proposed two-stage magnetic anomaly detection framework, beginning with the Orthonormal Basis Function detection principle, followed by Constant False Alarm Rate processing, and concluding with the novel hierarchical clustering approach featuring optimal cut height calculation.

### 2.1. Detection Framework Overview and OBF Principle

To eliminate the effects of environmental background magnetic noise in small target detection, vertical gradient detection is typically adopted. A vertical gradient detection system is also designed in this study. The proposed detection system employs a vertical gradient configuration with dual scalar magnetometers to effectively suppress large-scale geomagnetic background fluctuations. When magnetic targets possess small volumes or substantial burial depths, the induced magnetic anomaly amplitudes are typically weak and susceptible to being masked by background field variations. The vertical gradiometer system addresses this challenge by measuring the differential magnetic field between two vertically separated sensors.

**Figure 2:**
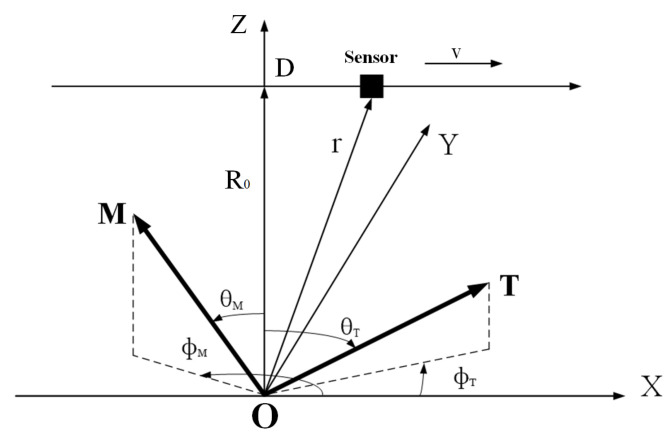
The coordinate system of relative motion between the magnetometer and the target.

To extract the target signal from the residual noise, we employ the detection algorithm based on Orthonormal Basis Function (OBF) decomposition, this detection algorithm is a widely used method for magnetic anomaly signal identification [21]. Based on the assumption that the target can be equivalently modeled as a magnetic dipole, this method represents the observed magnetic anomaly signal as a linear combination of three orthonormal basis functions. Figure 2 shows a Cartesian coordinate system established with the magnetic dipole as the origin, where the sensor moves along the *X*-direction at a velocity of *v* passing the target. Point *O* represents the target position, and $R_{0}$ denotes the minimum distance between the target and the magnetic sensor, as well as the distance between the magnetic dipole and the motion trajectory. $\theta_{T}$ and $\theta_{M}$ are the magnetic inclinations, representing the angles between the geomagnetic field *T*, the target magnetic moment *M* and the positive direction of the *Z*-axis, respectively. $\phi_{T}$ and $\phi_{M}$ are the magnetic declinations, representing the angles between the projections of the geomagnetic field *T* and the target magnetic moment *M* on the XY plane and the positive direction of the *x*-axis, respectively.

**Figure 3:**
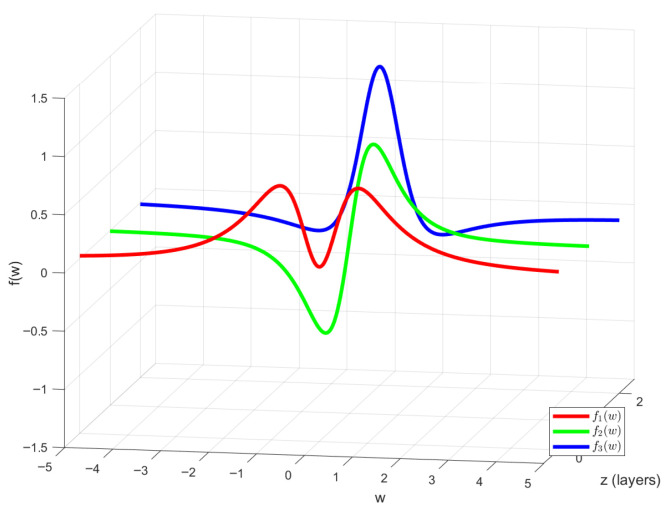
Three orthonormal basis function waveforms.

According to the magnetic dipole field formula, the magnetic field intensity at the sensor position can be calculated and transformed into a linear combination of three orthonormal basis functions $f_{1}\left(w\right),\;f_{2}\left(w\right),\;f_{3}\left(w\right)$ given by:

(1) $$\left\{\begin{array}{c} f_{1}\left(w\right)=\sqrt{\frac{128}{3\pi }}\frac{w^{2}}{{\left(1+w^{2}\right)}^{\frac{5}{2}}},\\ f_{2}\left(w\right)=\sqrt{\frac{128}{5\pi }}\frac{w}{{\left(1+w^{2}\right)}^{\frac{5}{2}}},\\ f_{3}\left(w\right)=\sqrt{\frac{24}{5\pi }}\frac{1-\frac{5}{3}w^{2}}{{\left(1+w^{2}\right)}^{\frac{5}{2}}}, \end{array}\right.$$

where $\omega =D/R_{0},\;D=vt$, representing a dimensionless coordinate along the magnetometer’s trajectory. Figure 3 displays the curves of the three orthonormal basis functions [21].

The signal expression can be transformed to obtain:

(2) $$S_{\mathrm{signal}\;}=\frac{\mu_{0}M}{4\pi R_{0}^{3}}\sum_{n=1}^{3}a_{n}f_{n}\left(w\right),$$

where $\mu_{0}$ is the permeability of free space and *M* is the magnetic moment, an energy function is formed by combining the coefficients $a_{n}$:

(3) $$E\left(m\right)=\sum_{n=1}^{3}a_{n}^{2}\left(m\right),$$

where *m* represents the current sampling point. Notably, the energy function can suppress the impact of the magnetic source’s magnetization direction on magnetic anomaly distribution: the magnetization direction only alters the distribution of the expansion coefficients $a_{1}$, $a_{2}$, $a_{3}$ in Equation (2) rather than the sum of their squares (the total signal energy), thus stably characterizing magnetic anomaly targets regardless of magnetization direction variations. In addition, this energy function has strong anti-interference characteristics owing to the matched projection of OBF decomposition: the target magnetic anomaly signal is highly concentrated on the three orthonormal basis functions in Equation (1) matching the magnetic dipole field characteristics, while random background noise energy is scattered in the entire function space and cannot form a significant energy peak, achieving effective separation of weak target signals and noise. This characteristic has been verified in classic magnetic anomaly detection research [21], and further supported by the stable weak signal extraction and reliable alarm point generation in the subsequent simulation (Section 3) and field experiments (Section 4). Consequently, the energy function can effectively extract weak magnetic anomaly signals from noise backgrounds, providing a reliable input for subsequent CFAR processing.

### 2.2. The Principle of Constant False Alarm Rate (CFAR) Detection

The energy function E(m) derived from the OBF decomposition, as described in the previous section, enhances the signal-to-noise ratio but still requires a robust decision mechanism to declare the presence of a target. Since the inherent randomness of background noise and clutter can cause false alarms, as the signal amplitude may exceed the detection threshold and be misidentified as a target even when no real target exists. To this end, the Constant False Alarm Rate (CFAR) detection algorithm is employed to adaptively set the detection threshold based on the local noise statistics, thereby maintaining a constant probability of false alarm [22,23]. CFAR technology adaptively calculates a detection threshold based on a pre-defined constant false alarm probability, enabling it to handle continuously varying signal amplitudes. This adaptive threshold dynamically adjusts to changes in background magnetic noise, thereby effectively highlighting target signals and enabling target identification.

**Figure 4:**
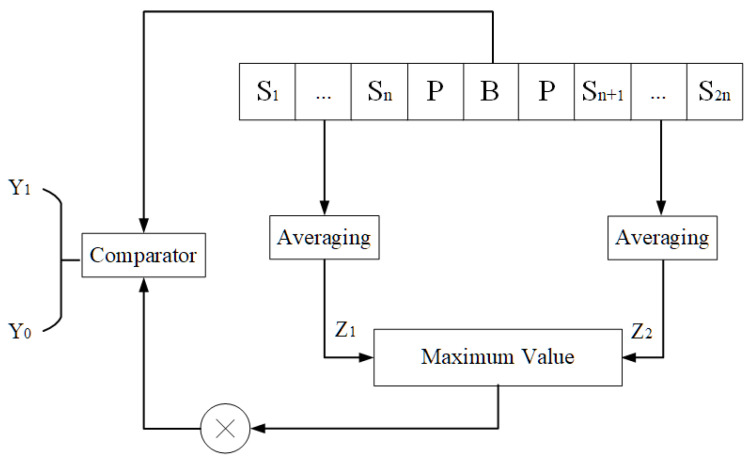
Greatest-of cell-averaging constant false alarm rate (GOCA-CFAR) detection.

Among various CFAR techniques, the Greatest-of Cell-Averaging CFAR (GOCA-CFAR) is particularly suited for magnetic anomaly detection in non-stationary noise environments. As illustrated in Figure 4, the GOCA-CFAR processor slides a window through the energy sequence E(m). For each Cell Under Test (CUT), the algorithm estimates the background noise power from leading and trailing reference windows. Guard cells are placed adjacent to the CUT to prevent contamination of the noise estimate by target energy. The algorithm then computes the average energy within each reference window (each containing *N* samples) and selects the greater of the two averages:

(4) $$Z=\max\left(\frac{1}{N}\sum_{i=1}^{N}E_{\mathrm{lead}\;}\left(i\right),\frac{1}{N}\sum_{j=1}^{N}E_{\mathrm{trail}\;}\left(j\right)\right).$$

This strategy provides robustness against sudden changes in background noise level at the boundaries of clutter regions. The adaptive threshold $T_{c}$ is then obtained by multiplying this noise estimate *Z* by a scale factor *c*:

(5) $$T_{c}=c\cdot Z.$$

The scale factor *c* is derived from the desired false alarm probability and the statistics of the noise [24]. The energy in the $E_{\mathrm{CUT}\;}$ is compared against this threshold $T_{c}$: if $E_{\mathrm{CUT}\;}>T_{c}$, a target is declared, and an alarm is generated; otherwise, the signal is classified as background noise.

In Figure 4, *B* denotes the CUT with the energy value of $E_{\mathrm{CUT}}$, and *P* represents the guard cells on its two sides. $S_{1}∼S_{n}$ and $S_{n+1}∼S_{2n}$ are the reference windows each containing *N* sampling points: the average energies $Z_{1}$ and $Z_{2}$ of the two reference windows are calculated separately, the larger of which is multiplied by the scale factor α, and the resulting value is then compared with $E_{\mathrm{CUT}}$. The signal $Y_{1}$ exceeds the threshold, indicating a detection, while $Y_{2}$ falls below it, corresponding to noise. This adaptive thresholding provided by GOCA-CFAR generates discrete alarm points that serve as the input for the spatial clustering stage described next.

### 2.3. Hierarchical Clustering with Optimal Cut Height

The one-dimensional OBF-CFAR detector processes data from each survey line independently, outputting discrete alarm points. A fundamental limitation of this approach is its inherent inability to distinguish between a spatially isolated false alarm and a coherent cluster of alarms generated by a single physical target. In a two-dimensional plane, it is easier to correlate these alarm points and estimate the target’s geographic center.

**Figure 5:**
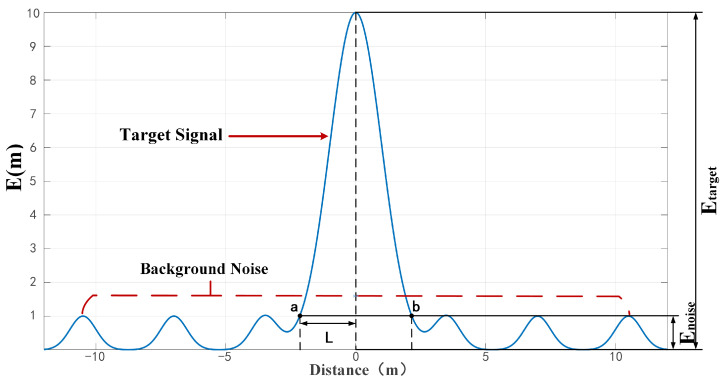
The target signal’s detectable range within background noise.

The spatial distribution characteristics of these alarm points are illustrated in Figure 5. A magnetic anomaly trigger alarms within its effective detection range (interval a-b in the figure). The underlying mechanism can be explained by four key aspects: (1) Distance Dependency: When the sensor is close to the target (horizontal distance d<l), the amplitude of the energy $E_{target}$ derived from the anomalous magnetic field $B_{target}$ generated by the target exceeds the energy $E_{noise}$ calculated from the background noise threshold $B_{noise}$. (2) Detection Boundary: As the sensor moves away from the target (d>l), $E_{target}$ attenuates below $E_{noise}$ and the signal becomes drowned by the background noise. (3) Valid Alarm Range: Alarm signals are considered valid only within the interval a-b, where $E_{target}$ consistently exceeds $E_{noise}$. (4) Spatial Correlation of Discrete Signals: Multiple survey lines passing near a target form a spatially clustered set of alarm points centered around it. Signals falling within the magnetic anomaly coverage range near the target can be considered valid detections. Hierarchical clustering is suitable for scenarios with an unknown number of targets, as it effectively aggregates discrete alarm points originating from the same source. This makes it particularly useful for false alarm suppression in unexploded ordnance (UXO) detection.

Hierarchical Clustering is a tree-structured clustering method that progressively merges or splits data points to form a dendrogram, visually revealing the intrinsic similarity within the data [25]. During the clustering process, data points with high similarity are gradually aggregated into smaller clusters, which are then merged into larger clusters, eventually forming a hierarchical tree-like structure. This dendrogram allows for an intuitive determination of clustering results at different scales, enabling the selection of an appropriate classification outcome based on practical requirements. Since this method does not require a pre-defined number of clusters, it is suitable for magnetic detection scenarios where the number of targets is unknown. Moreover, it exhibits strong robustness in handling noise and outliers, effectively avoiding severe disruptions to the clustering results caused by individual anomalous or noisy data points. Therefore, it is well-suited for magnetic anomaly detection, where measurement noise and outliers are frequently encountered.

**Figure 6:**
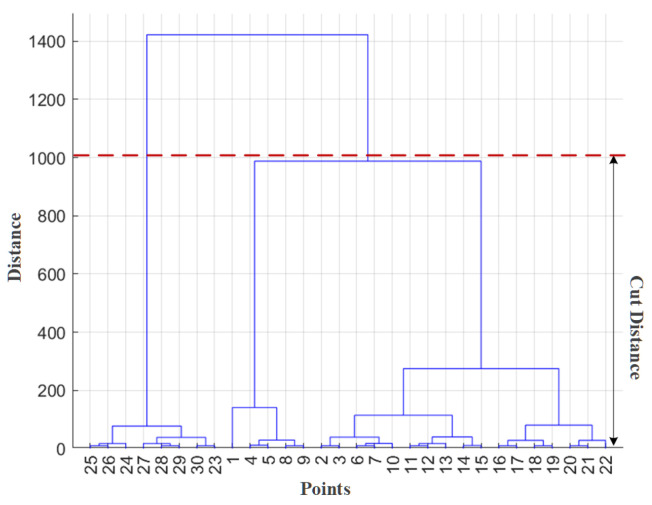
Dendrogram of hierarchical clustering.

To minimize the introduction of false alarms during magnetic anomaly detection, the Complete Linkage cluster merging strategy is adopted. This strategy uses the maximum distance between any two points in two clusters, denoted as $D\left(C_{i},\;C_{j}\right)$, as the criterion for merging clusters:

(6) $$D\left(C_{i},\;C_{j}\right)=\max_{a\in C_{i},\;b\in C_{j}}d\left(a,\;b\right),$$

where d(a, b) represents the distance between point *a* in cluster $C_{i}$ and point *b* in cluster $C_{j}$. After two clusters are merged into a new cluster, the algorithm continues to search for the farthest point between this new cluster and another cluster for the next merger. This process repeats until all points within the survey area are merged into a single cluster. In the context of magnetic anomaly detection and localization, the positions of anomaly points are described in Euclidean space, making the Euclidean distance—which aligns with conventional geometric intuition—a suitable choice for calculating point-to-point distances. A dendrogram generated by hierarchical clustering is shown in Figure 6. The leaf nodes at the bottom represent the measured anomaly points. As the distance threshold increases, more points or clusters merge step by step, ultimately forming a single cluster.

Based on the survey line spacing and the cut height, the expected count of alarm points within a cluster generated by the target signal can be calculated. Clusters containing fewer than half of the expected number of alarm points are classified as false alarms. The cut height $d_{\mathrm{cut}}$ is a critical parameter in the hierarchical clustering algorithm. During the algorithm’s execution, the merging distance between clusters—calculated based on the selected cluster update strategy and distance metric—is compared against $d_{\mathrm{cut}}$. The clustering process terminates when the merger distance exceeds the cut height. Currently, the cut height is typically preset empirically as an input parameter, which can significantly influence the final clustering outcome.

To resolve this critical issue, the core contribution of this work is a novel, physics-based method for determining the optimal cut height directly from the acquired magnetic signals, eliminating the need for empirical guesswork. The following section details the theoretical derivation of this optimal $d_{\mathrm{cut}}$.

The spatial distribution of alarm points from magnetic anomaly targets is constrained by their effective detection range. As shown in Figure 5, alarm points are generated only when the sensor is within distance *L* from the target and the signal exceeds the noise floor. Isolated alarms outside this range are considered false alarms. Therefore, the optimal cut height for clustering should relate to 2L, which depends on target magnetic moment, sensor height, and background noise level.

(7) $$\Delta B_{z}\left(L,\alpha ,\beta \right)=\frac{\mu_{0}m}{4\pi }\left[F\left(L,z_{\mathrm{low}},\alpha ,\beta \right)-F\left(L,z_{\mathrm{high}},\alpha ,\beta \right)\right].$$

**Figure 7:**
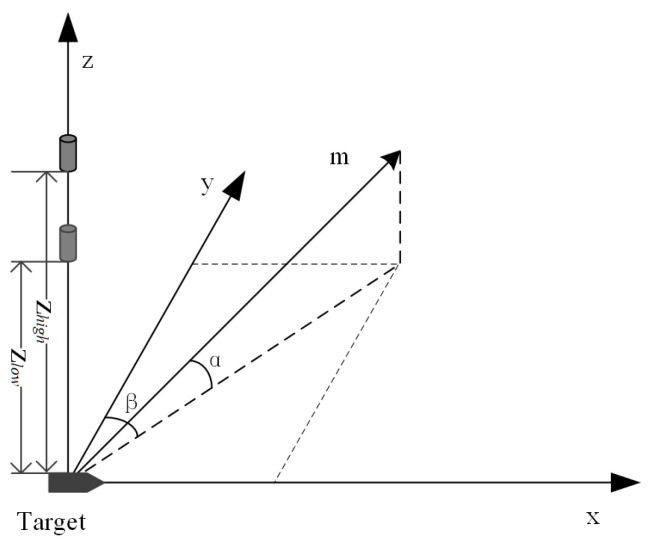
Schematic diagram of magnetic detection using a vertical gradiometer.

In scenarios detecting magnetic anomaly targets using optically pumped magnetometers under vertical gradient configuration, as illustrated in Figure 7, a coordinate system is established with the magnetic dipole as the origin. The effective detection range for a magnetic anomaly target is defined as the maximum horizontal distance $L_{\max}$ between the magnetometer and the target when the vertical gradient magnetic anomaly generated by the dipole attenuates to $B_{\mathrm{noise}}$, where $B_{\mathrm{noise}}$ includes both the environmental background noise and magnetic anomaly signals produced by non-target bodies. This detection range boundary serves as the fundamental criterion for distinguishing genuine targets from false alarms. Alarm points generated within this $L_{\max}$ radius around a target correspond to valid magnetic anomaly signals, while isolated detections beyond this range are considered false alarms caused by background noise fluctuations. The optimal cut height $d_{cut}=2L_{\max}$ is therefore designed to ensure that all valid alarm points from a single target are grouped together while excluding spatially isolated false alarms.

In practical magnetic anomaly detection, the orientation of the target’s magnetic moment significantly affects the signal strength. To ensure the most conservative (i.e., largest) estimate of the detection range, it is necessary to calculate the magnetic moment orientation that maximizes this range.

In a vertical magnetic gradiometer system, the signal strength received by the sensor depends on the projection of the target’s magnetic moment on the Earth’s magnetic field (EMF) direction. The magnetic moment orientation can be described by two angles: the angle α between the magnetic moment and the vertical direction, and the angle β between the horizontal component of the magnetic moment and the horizontal component of the geomagnetic field.

When the horizontal component of the magnetic moment aligns with the horizontal component of the geomagnetic field, i.e., β=0°, the interaction between the target’s magnetic moment and the geomagnetic field is strongest. This is due to the magnetization effect of the geomagnetic field on ferromagnetic targets, causing their induced magnetic moment to align with the geomagnetic field direction. In practical scenarios, ferromagnetic targets such as unexploded ordnance typically exhibit a significant magnetization component along the geomagnetic field direction. Therefore, β=0° corresponds to the case where the target’s magnetic moment generates the strongest detection signal.

Therefore, when solving for the maximum detection range, we first set β=0°, simplifying the problem to single-variable optimization focused on finding the optimal inclination angle $\alpha^{*}$. By considering the orientation that maximizes the detection range, we ensure that the calculated cut height provides sufficient coverage even for targets with suboptimal magnetic moment orientations, thereby enhancing the algorithm’s robustness in practical applications where target orientation is unknown.

The numerical solution for $L_{\max}$ using the Newton-Raphson method demonstrates good convergence properties, typically reaching the desired precision within a few iterations. The algorithm demonstrated good numerical stability across the parameter ranges encountered in practical magnetic anomaly detection scenarios.

To ensure clustering integrity, we adopt a conservative design principle: we assume that the target’s magnetic moment is oriented in the direction that yields the maximum possible detection range. This approach guarantees that even if the actual magnetic moment direction is unknown or varies, the algorithm maintains sufficient redundancy to encompass all potential valid alarm points.

For a vertical gradiometer configuration with sensors at heights $z_{\mathrm{low}}$ and $z_{\mathrm{high}}$, the differential signal based on magnetic dipole theory can be expressed as a function of horizontal distance *L* and magnetic moment inclination α:

(8) $$\Delta B_{T}\left(L\right)=\frac{\mu_{0}M}{4\pi }\left[\frac{N(z_{\mathrm{low}},\alpha )}{{\left(L^{2}+z_{\mathrm{low}}^{2}\right)}^{3/2}}-\frac{N(z_{\mathrm{high}},\alpha )}{{\left(L^{2}+z_{\mathrm{high}}^{2}\right)}^{3/2}}\right],$$

where N(z,α) represents the direction-dependent function incorporating the geometric relationships between sensor height, magnetic moment inclination α, and geomagnetic inclination *I*.

The detection range $L_{\max}$ is mathematically defined as satisfying $\Delta B_{T}\left(L_{\max},\alpha \right)=B_{\mathrm{noise}}$. Since $L_{\max}$ is an implicit function of α, we employ implicit differentiation to identify the magnetic moment direction that maximizes the detection range. Differentiating the boundary condition with respect to α using the chain rule:

(9) $$\frac{\partial }{\partial \alpha }\left[\Delta B_{T}\left(L_{\max},\alpha \right)\right]=\frac{\partial \Delta B_{T}}{\partial L_{\max}}\cdot \frac{\partial L_{\max}}{\partial \alpha }+\frac{\partial \Delta B_{T}}{\partial \alpha }=0.$$

At the extremum point $\alpha =\alpha^{*}$, the detection range becomes insensitive to variations in magnetic moment direction ($\partial L_{\max}/\partial \alpha =0$), simplifying the extremum condition to:

(10) $$\frac{\partial \Delta B_{T}}{\partial \alpha }=0,$$

calculating the partial derivative of the direction function N(z,α) yields:

(11) $$\frac{\partial N(z,\alpha )}{\partial \alpha }=A\left(z\right)\sin\alpha +B\left(z\right)\cos\alpha ,$$

where the coefficient functions are defined as:

(12) $$\begin{array}{cc} A(z) & =-3L\left(L\cos I+z\sin I\right)+\left(L^{2}+z^{2}\right)\cos I, \end{array}$$

(13) $$\begin{array}{cc} B(z) & =3z\left(L\cos I+z\sin I\right)-\left(L^{2}+z^{2}\right)\sin I. \end{array}$$

Expanding the partial derivative of $\Delta B_{T}$ leads to a new extremum condition:

(14) $$D\left(z_{\mathrm{low}\;}\right)\cdot \frac{\partial N\left(z_{\mathrm{low}\;},\alpha \right)}{\partial \alpha }-D\left(z_{\mathrm{high}\;}\right)\cdot \frac{\partial N\left(z_{\mathrm{high}\;},\alpha \right)}{\partial \alpha }=0.$$

Substituting into the extremum condition and solving through algebraic transformation yields the analytical solution for optimal magnetic moment inclination:

(15) $$\alpha^{*}=\arctan\left(\frac{\frac{B(z_{\mathrm{high}})}{{\left(L^{2}+z_{\mathrm{high}}^{2}\right)}^{3/2}}-\frac{B(z_{\mathrm{low}})}{{\left(L^{2}+z_{\mathrm{low}}^{2}\right)}^{3/2}}}{\frac{A(z_{\mathrm{low}})}{{\left(L^{2}+z_{\mathrm{low}}^{2}\right)}^{3/2}}-\frac{A(z_{\mathrm{high}})}{{\left(L^{2}+z_{\mathrm{high}}^{2}\right)}^{3/2}}}\right).$$

After determining the optimal magnetic moment direction $\alpha^{*}$, the maximum detection radius $L_{\max}$ is obtained by numerically solving the constraint equation $\Delta B_{T}\left(L_{\max},\alpha^{*}\right)=B_{\mathrm{noise}}$ using methods such as Newton-Raphson iteration. The optimal cut height for hierarchical clustering is then established as:

(16) $$d_{cut}=2L_{\max}.$$

This selection ensures that all alarm points within the effective detection range of radius $L_{\max}$ centered on the target are correctly clustered together. Since $L_{\max}$ is a conservative estimate based on the maximum detection range, the resulting cut height provides sufficient adaptability to the actual variations in the direction (azimuth and inclination) of the target’s magnetic moment, while theoretically guaranteeing clustering integrity.

The optimal cut height obtained through this physics-based calculation ensures that all alarm points generated by magnetic anomaly targets are aggregated into single clusters while effectively excluding false alarms caused by non-target sources. This theoretical model provides a direct, principled method for calculating the optimal cut height, eliminating the empirical guessing that has traditionally limited clustering-based detection methods.

## 3. Simulation and Results

### 3.1. Experimental Setup and Single-Group Analysis

The simulation experiments were designed to provide a comprehensive performance assessment of the proposed method, aiming to validate the theoretical optimal cut height model while quantifying its false alarm suppression capability. The evaluation encompasses comparisons with both the traditional OBF-CFAR detection approach and clustering methods employing non-optimal cut heights, thereby demonstrating the critical importance of the theoretically derived parameter in achieving robust target detection.

The process first simulated the geomagnetic background field by defining a 20 m × 20 m area. To create a realistic magnetic background, magnetic dipole signals from 200 sources with randomized positions, burial depths, and magnetic moment orientations were incorporated, along with superimposed random trend variations. Additionally, magnetic dipole signals from three targets at random locations were embedded in the region, each with a magnetic moment of 0.8 A·m^2^ at 0.5 m depth. Then simulated a gradiometer system with two sensors mounted at heights of 0.8 m and 1.6 m above the ground, respectively, conducting a survey over the area with a line spacing of 0.2 m to obtain the vertical magnetic gradient distribution data.

**Figure 8:**
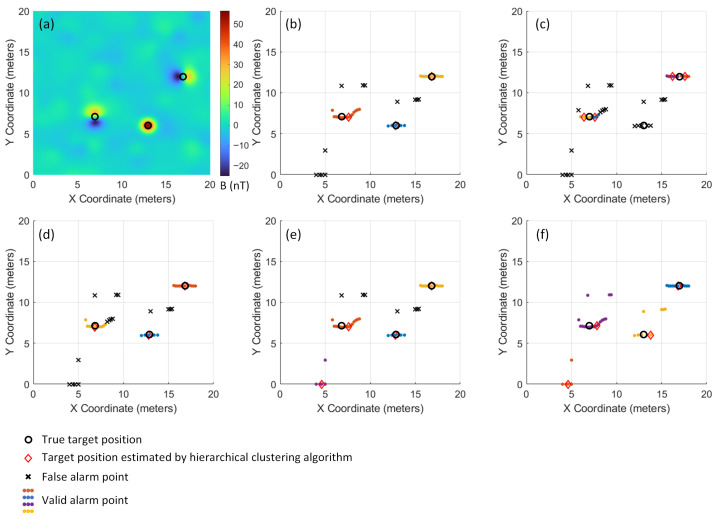
Comparison of clustering effects under different cut height settings. (**a**) Original magnetic anomaly signal distribution (gridded by linear interpolation); (**b**) Optimal cut height accurately identifies three targets while excluding false alarms; (**c**) 0.5 times of the cut height causes target signal fragmentation; (**d**) 0.75 times of the cut height results in the misjudgment of the valid alarm points as false alarm points; (**e**) 1.5 times of the cut height introduces false alarm misjudgments; (**f**) 2 times of the cut height leads to severe false alarm merging.

To demonstrate the clustering effects under different cut height settings, we selected a representative dataset for detailed analysis. The experiments employed the theoretically calculated optimal cut height, as well as 0.5, 0.75, 1.5, and 2 times of this optimal value $2L_{max}$ for hierarchical clustering. As shown in Figure 8, Figure 8a displays the distribution of the original magnetic anomaly signals. After clustering with the optimal cut height parameter, Figure 8b clearly distinguishes the alarm point clusters corresponding to three targets through different colors, while effectively eliminating isolated false alarms. Figure 8c,d show the clustering results with 0.5 times and 0.75 times of the optimal cut height, respectively. It can be observed that due to the excessively small cut height settings, a single genuine target signal is incorrectly fragmented into multiple clusters, and some genuine target signals are misclassified as false alarms. Figure 8e,f present the clustering results with 1.5 times and 2 times the optimal cut height, where the overly large cut height settings cause some false alarm signals to be erroneously merged into the target clusters, resulting in false alarm misjudgments.

### 3.2. Multi-Group Robustness Validation

To comprehensively assess the performance of the proposed method, we conducted extensive simulations across nine independent datasets with varying target configurations and noise characteristics. The optimal cut height algorithm was applied individually to each dataset to calculate the corresponding cut height $d_{\mathrm{cut}}$. Hierarchical clustering was then employed to cluster the alarm points and identify false alarms.

**Figure 9:**
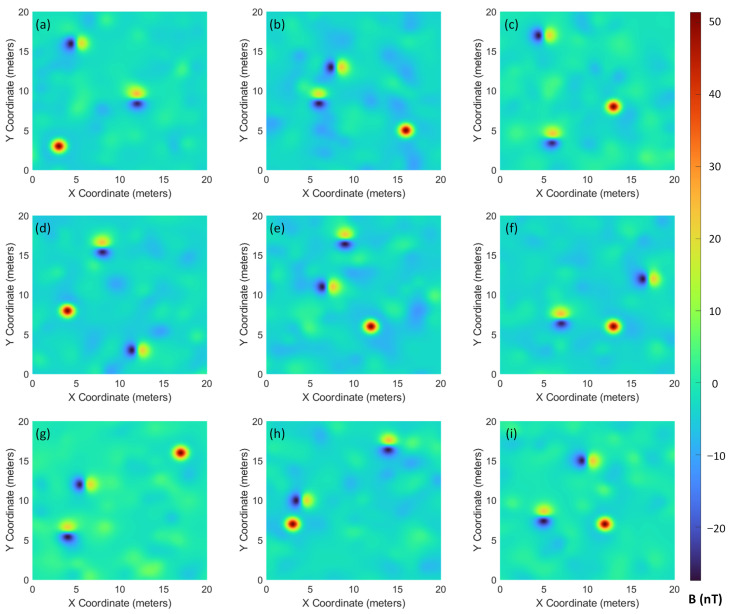
Magnetic field distribution maps from nine simulated datasets with varying target configurations and noise characteristics. (**a**) Magnetic field distribution map of the first simulation dataset; (**b**) Magnetic field distribution map of the second simulation dataset; (**c**) Magnetic field distribution map of the third simulation dataset; (**d**) Magnetic field distribution map of the fourth simulation dataset; (**e**) Magnetic field distribution map of the fifth simulation dataset; (**f**) Magnetic field distribution map of the sixth simulation dataset; (**g**) Magnetic field distribution map of the seventh simulation dataset; (**h**) Magnetic field distribution map of the eighth simulation dataset; (**i**) Magnetic field distribution map of the ninth simulation dataset.

**Figure 10:**
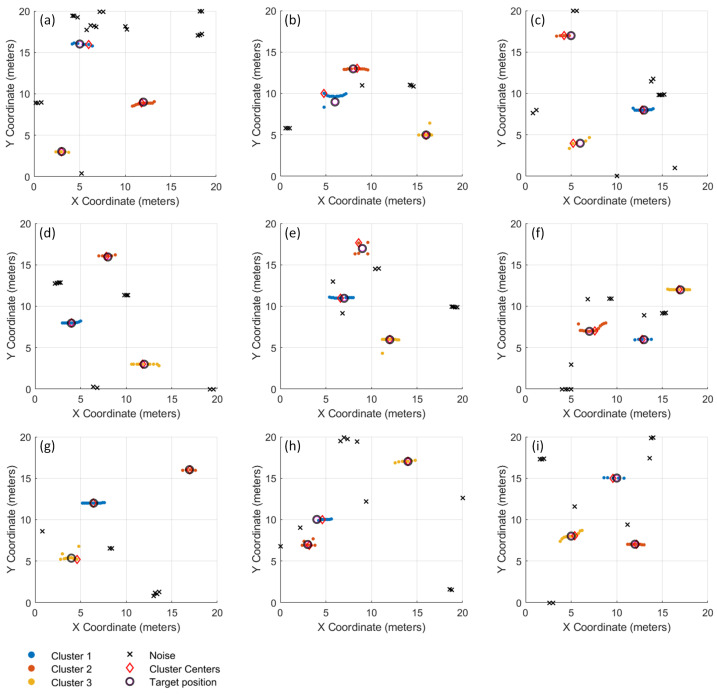
Alarm information from the nine simulation datasets processed by the optimal cut height clustering algorithm. (**a**) Alarm information of the first simulation dataset; (**b**) Alarm information of the second simulation dataset; (**c**) Alarm information of the third simulation dataset; (**d**) Alarm information of the fourth simulation dataset; (**e**) Alarm information of the fifth simulation dataset; (**f**) Alarm information of the sixth simulation dataset; (**g**) Alarm information of the seventh simulation dataset; (**h**) Alarm information of the eighth simulation dataset; (**i**) Alarm information of the ninth simulation dataset.

The resulting magnetic field distribution maps generated from the nine datasets are shown in Figure 9, while the corresponding clustering results are presented in Figure 10. Analysis demonstrates the algorithm’s effectiveness in discriminating between true targets and false alarms while consolidating spatially related signals. Across the nine simulation trials, the method reliably identified all three embedded targets in eight experiments, with only a single missed detection in trial Figure 10g.

Based on the final results, statistics of alarm points and false alarms were compiled. Table 1 shows the clustering results under different independent simulation scenarios, the proportion of eliminated false alarms for each dataset can be observed. In some cases, false alarm points located within the coverage area of the target signals were mistakenly identified as valid alarm points, as seen in Figure 10b,e,f. Since these alarm points all appear within the target signal coverage area, this situation may affect more refined localization but does not compromise target identification.

Subsequently, we conducted comparisons using 0.5 times and 2 times of the optimal cut height on nine datasets, and the statistical results are presented in Table 2. As summarized in Table 2, the performance metrics reveal substantial degradation with non-optimal cut heights. The algorithm using the optimal cut height achieved superior performance with 98% correct alarm classification and 96.3% target recognition accuracy. In contrast, the 0.5 times of the optimal cut height configuration yielded only 88.3% correct alarm classification and 70.4% target recognition, while the 2.0 times of the configuration achieved 86.9% and 81.5% respectively. These results demonstrate that both undersized and oversized cut heights significantly compromise performance—the former by fragmenting true targets into multiple clusters, and the latter by merging false alarms with legitimate targets.

## 4. Field Experiments and Analysis

### 4.1. Experimental Setup and Objectives

To validate the performance of the proposed magnetic anomaly detection algorithm based on hierarchical clustering with optimal cut height under real-world conditions, we conducted a field experiment using UXO models. The experiment utilized a gradiometer system constructed with two cesium optically pumped magnetometers developed by Peking University. The magnetometers have a noise spectral density of $0.3\;\mathrm{pT}/\sqrt{\mathrm{Hz}}$ at 1 Hz and a sampling rate of 10 Hz [26]. A GPS receiver with Real-Time Kinematic (RTK) capability was used for positioning, providing horizontal accuracy within 2 cm. Key specifications of the measurement system are summarized in Table 3.

**Figure 11:**
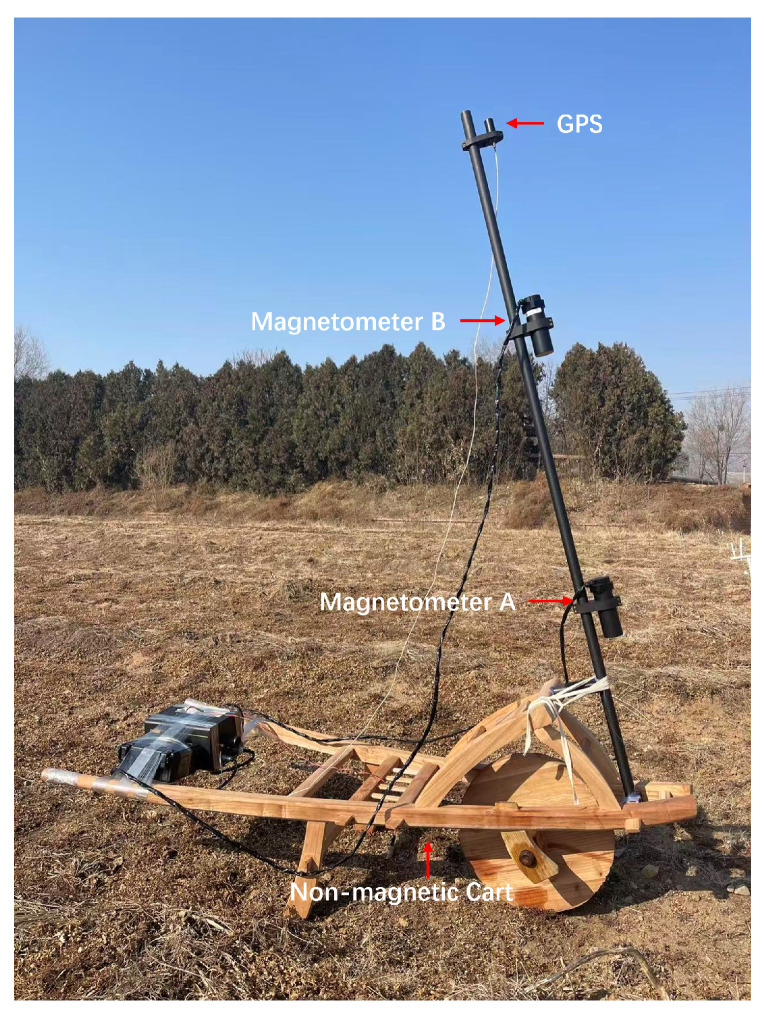
The vertically configured optically pumped magnetometer gradiometer system mounted on a non-magnetic cart.

Magnetometer A and Magnetometer B were mounted at heights of 0.8 m and 1.6 m above the ground, respectively, on a vertical non-magnetic carbon fiber pole, as shown in Figure 11. The pole was securely fixed to a non-magnetic cart to effectively minimize the impact of mechanical vibrations on the magnetometers. A GPS receiver installed at the top of the pole.

**Figure 12:**
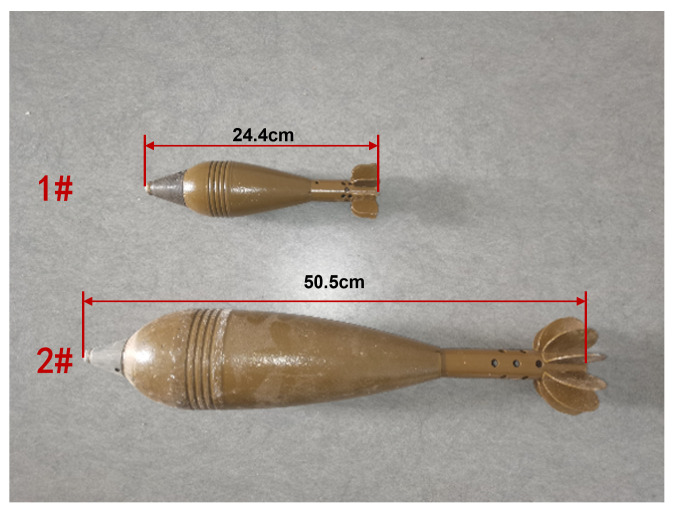
The unexploded ordnance (UXO) model.

The experiment was conducted on a sufficiently flat terrain to ensure operational feasibility. A survey area of 10 m × 10 m was established, with survey lines spaced at 1 m intervals, resulting in a total of 11 parallel lines across the area. The two targets, 1 # and 2 #, with lengths of 24.4 cm and 50.5 cm respectively, as shown in Figure 12, were buried at two locations at a depth of 0.1 m. The precise coordinates of the center points of the buried UXO models were recorded using GPS, after which the site survey was carried out. The data acquisition and processing followed a systematic pipeline:

1. **Data Collection**: The gradiometer system was moved along predetermined survey lines at walking speed (1 m/s), collecting vertical magnetic gradient data synchronized with GPS positioning.
2. **Preprocessing**: Raw data underwent bandpass filtering (0.1–5 Hz) to remove high-frequency noise and low-frequency drift while preserving the target signal bandwidth.
3. **OBF-CFAR Detection**: The filtered data was processed using the OBF decomposition method with a GOCA-CFAR detector (false alarm probability $P_{\mathrm{fa}}=10^{-3}$), generating discrete alarm points along each survey line.
4. **Survey Line Densification**: Due to the sparse original line spacing (1 m), linear interpolation was applied to create virtual survey lines at 0.14 m intervals. This densification provided sufficient spatial sampling for effective clustering while maintaining computational efficiency.
5. **Optimal Cut Height Calculation**: The algorithm calculated an optimal cut height of $d_{cut}=2.42$ m based on measured parameters: peak gradient signal was 112.3 nT, background noise was 10.2 nT, and local geomagnetic inclination was 59°.
6. **Hierarchical Clustering**: Complete-linkage hierarchical clustering was applied using the calculated $d_{\mathrm{cut}}$, grouping spatially related alarm points into target clusters.

### 4.2. Analysis of Experimental Results

**Figure 13:**
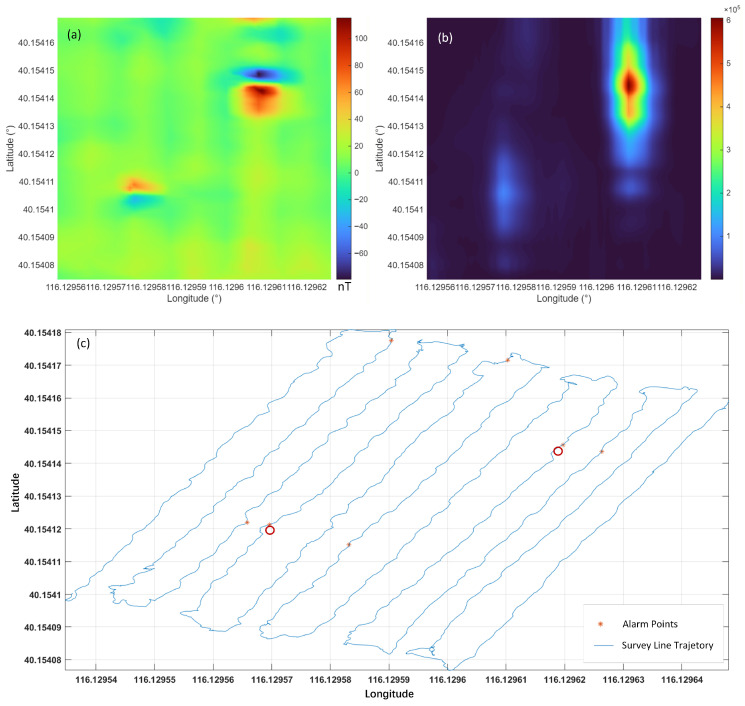
Magnetic Anomaly Detection Results Based on the CFAR-OBF Algorithm. (**a**) The original magnetic anomaly map; (**b**) The energy function distribution map; (**c**) Detection results of the CFAR-OBF algorithm. Red circles represent the buried positions of targets, and red asterisks denote the alarm points detected by the OBF detection algorithm.

First, classic orthonormal basis function-based constant false alarm rate detection and localization were performed on the survey data. After applying filtering procedures to the data collected by the gradiometer, the signal energy was computed using one-dimensional orthonormal basis functions and compared against the threshold set by the CFAR detector. Alarm points were then extracted and their geographic coordinates (longitude and latitude) were recorded. As shown in Figure 13, a total of seven alarm points were identified. Comparing these with the burial locations marked by red circles in the figure, it can be observed that two of the alarm points are closely spaced, suggesting that they correspond to the two buried UXO targets. While two clusters of points were visually apparent near the known target locations, the method could not automatically distinguish these from isolated false alarms, requiring manual interpretation.

Following the optimal cut height calculation method outlined in Section 2.3, the peak-to-peak gradient signal of the survey line data was first calculated to be 112.3 nT, and the noise level across the entire survey area was determined to be 10.2 nT. Based on the latitude and longitude coordinates of the measurement site, the local geomagnetic inclination was estimated to be approximately 59°.

The calculation yielded an optimal magnetic moment inclination angle of approximately α≈87.2° relative to the vertical direction, which maximizes the detection range for the magnetic target. Based on parameters including the heights of the two gradiometers above ground, signal amplitude, noise level, and local magnetic inclination angle, the maximum effective detection radius was calculated as $L_{\max}$ = 1.21 m, resulting in an optimal cut height of $2L_{\max}$ = 2.42 m.

**Figure 14:**
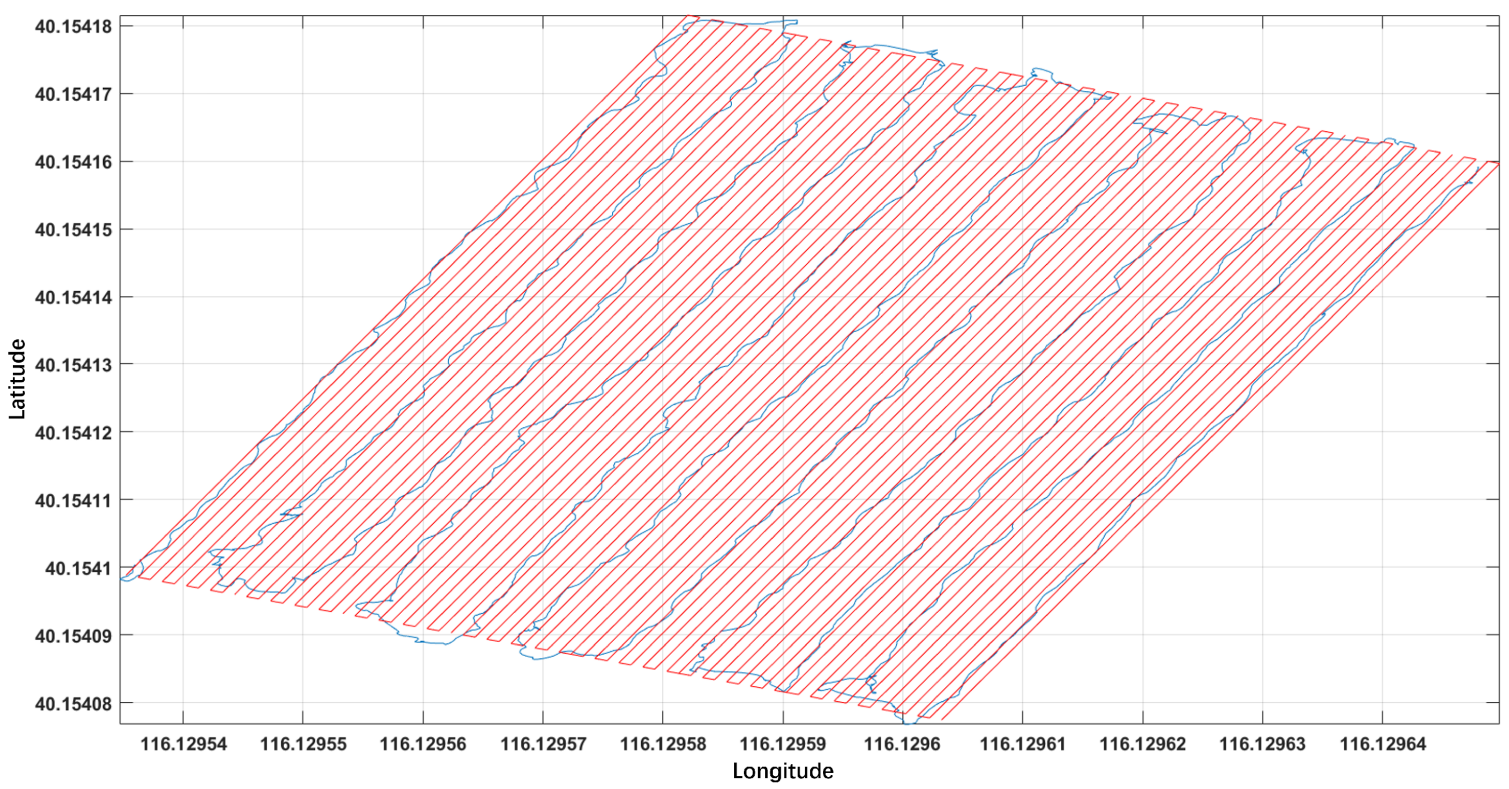
Survey lines with a densified spacing of 0.14 m after processing. Blue lines denote magnetometer-measured trajectories, and red lines denote virtual survey lines generated via survey line densification.

Due to the low density of the original survey lines, linear interpolation was applied to the survey line data to generate a two-dimensional magnetic anomaly distribution map and provide sufficient alarm points for the clustering algorithm. In the field experiment, the measurement system was mounted on a non-magnetic cart pushed manually with a moving speed of approximately 5 km/h, and the interval of 0.14 m was determined according to the actual physical sampling interval corresponding to the system sampling frequency and the cart moving speed. The survey area was densified with such an interpolation spacing. The processed results are shown in Figure 14.

**Figure 15:**
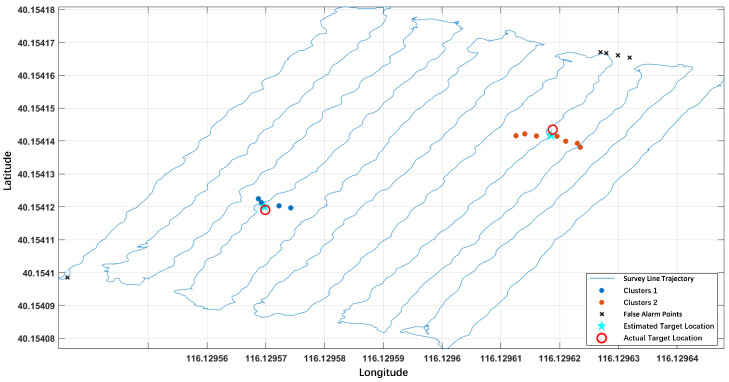
Clustering results with 0.14 m line spacing.

After survey line densification and application of the proposed clustering algorithm, the results demonstrated significant improvement. As shown in Figure 15, the clustering algorithm successfully identified two valid clusters, marked in blue and red points. The calculated optimal cut height effectively distinguishes valid alarm points from false alarms. The target locations were estimated based on the geometric centers of the alarm points in the two clusters.

The field experiment results demonstrate the practical effectiveness of the proposed algorithm. Two key observations emerge from the analysis:

**Optimal Cut Height Validation**: The calculated $d_{cut}=2.42$ m proved appropriate for the field conditions, successfully grouping all alarm points from each target while excluding environmental false alarms. This validates the theoretical model’s applicability to real-world scenarios.

**Operational Efficiency Improvement**: The clustering algorithm reduced the number of locations requiring manual verification from 7 to 2, representing a 71% reduction in field investigation effort. This efficiency gain is particularly valuable in UXO clearance operations where each potential target requires time-consuming physical inspection.

Despite these considerations, the field experiment successfully demonstrates that the proposed algorithm provides a systematic approach for transforming OBF-CFAR detection outputs into actionable intelligence, significantly enhancing the practicality of magnetic anomaly detection for UXO clearance operations.

## 5. Conclusions

This study has successfully addressed the critical challenge of high false alarm rates in magnetic anomaly detection by developing a novel two-stage framework that integrates OBF-CFAR detection with optimal cut height-based hierarchical clustering. The proposed method effectively overcomes the limitations of traditional one-dimensional approaches by exploiting the spatial coherence of genuine magnetic anomalies across multiple survey lines, achieving over 96.3% target recognition accuracy in simulation experiments and 100% accuracy in field experiments while significantly reducing false alarms.

The core innovation lies in establishing a theoretical model that calculates the optimal cut height for hierarchical clustering directly from physical parameters, which solves the problem that traditional hierarchical clustering relies on manual empirical judgment to determine the critical parameter, eliminating empirical parameter guesswork and ensuring robust false alarm suppression. Both simulation and field validation confirm the algorithm’s capability to reliably distinguish target clusters from random noise, providing a practical solution for UXO detection and similar applications. Future work will focus on developing pattern recognition techniques based on magnetic anomaly signature modeling to further enhance discrimination capability for closely spaced multiple targets.

## Figures and Tables

**Table 1:** Statistics of False Alarm Identification Rate in Simulation Experiments.

No.	Cut Height (m)	Alarm Points	Identified False Alarms	Unidentified False Alarms	False Alarm Identification Rate	Signal Recognition Accuracy Rate
a	2.134	45	20	0	44.4%	100.0%
b	2.278	42	7	1	16.7%	97.6%
c	2.287	34	12	0	35.3%	100.0%
d	2.436	38	11	0	28.9%	100.0%
e	2.117	37	8	1	21.6%	97.3%
f	2.653	45	12	1	26.7%	97.8%
g	2.094	39	15	4	38.5%	90.0%
h	1.961	33	10	0	30.3%	100.0%
i	2.157	38	10	0	26.3%	100.0%

**Table 2:** Comparative performance with different cut heights (A total of 351 alarm points were detected by OBF-CFAR).

Configuration	Correct Points	Signal Recognition Accuracy Rate	Incorrect Target Identification Times	Target Recognition Accuracy Rate
Optimal Cut Height	344	98%	1	96.3%
0.5 × Optimal Cut Height	310	88.3%	8	70.4%
2 × Optimal Cut Height	305	86.9%	5	81.5%

**Table 3:** Specifications of the Magnetic Measurement System.

Parameter	Specification
Magnetometer type	Cesium optically pumped
Noise density (@1 Hz)	$0.3\;\mathrm{pT}/\sqrt{\mathrm{Hz}}$
Sampling rate	10 Hz
Sensor heights	0.8 m/1.6 m
GPS accuracy (RTK)	<2 cm

## Data Availability

The raw magnetic survey data are not publicly available due to ongoing research involving patent applications and commercial sensitivity, as well as privacy and security concerns regarding the precise geographic location of the survey sites. Processed results are fully presented in the manuscript. Researchers may contact the corresponding author to discuss potential access under a data transfer agreement.
